# A Cultural Evolution Approach to Digital Media

**DOI:** 10.3389/fnhum.2016.00636

**Published:** 2016-12-15

**Authors:** Alberto Acerbi

**Affiliations:** School of Innovation Science, Eindhoven University of TechnologyEindhoven, Netherlands

**Keywords:** cultural evolution, cultural transmission, transmission biases, cultural attraction, digital media, social media

## Abstract

Digital media have today an enormous diffusion, and their influence on the behavior of a vast part of the human population can hardly be underestimated. In this review I propose that cultural evolution theory, including both a sophisticated view of human behavior and a methodological attitude to modeling and quantitative analysis, provides a useful framework to study the effects and the developments of media in the digital age. I will first give a general presentation of the cultural evolution framework, and I will then introduce this more specific research program with two illustrative topics. The first topic concerns how cultural transmission biases, that is, simple heuristics such as “copy prestigious individuals” or “copy the majority,” operate in the novel context of digital media. The existence of transmission biases is generally justified with their adaptivity in small-scale societies. How do they operate in an environment where, for example, prestigious individuals possess not-relevant skills, or popularity is explicitly quantified and advertised? The second aspect relates to fidelity of cultural transmission. Digitally-mediated interactions support cheap and immediate high-fidelity transmission, in opposition, for example, to oral traditions. How does this change the content that is more likely to spread? Overall, I suggest the usefulness of a “long view” to our contemporary digital environment, contextualized in cognitive science and cultural evolution theory, and I discuss how this perspective could help us to understand what is genuinely new and what is not.

## 1. Introduction

Digital media are media encoded in digital format, typically to be transmitted and consumed on electronic devices, such as computers and smartphones. Digital media of wide diffusion includes emails, digital audio and video recordings, ebooks, blogs, instant messaging, and more recently social media. Although, digital media started to be developed with the creation of digital computers in the 1940s, their wide cultural impact can be traced back only to two or three decades, with the widespread diffusion of personal computers and especially the internet (Briggs and Burke, 2009).

Social media and ubiquitous connectivity (e.g., allowed by portable digital devices) are even more recent developments. Facebook, in its early stage limited to university or high-school students and employees of a handful of companies, was open to the public 10 years ago, in September 2006 (Boyd and Ellison, 2007). The first version of the iPhone, which gave the initial momentum to the worldwide diffusion of smartphones, was launched shortly after, at the beginning of 2007 (West and Mace, 2010).

Despite that, digital media, and social media in particular, have today an enormous reach. Facebook for example counts, as of June 2016, more than 1.7 billion monthly active users^1^. The influence of digital media on the behavior of a vast part of the human population is unanimously recognized. As a consequence, academic interest for digital media has grown rapidly in different disciplines. Here, I will not attempt a review of the existing literature, but I will propose that a specific scientific field, cultural evolution, could provide a suitable framework to analyse how the massive diffusion of digital media influences human cultural behavior.

The article is structured as follows. In the next section I will provide a brief and general introduction to the field of cultural evolution, focusing on the aspects I consider more relevant for the study of contemporary digital media. These aspects are cultural evolution's naturalistic and quantitative approach and its commitment to develop hypotheses informed by cognitive science and evolutionary theory. I will then explore more in depth two areas of research where cultural evolution could give an original contribution. First, I will discuss how cultural transmission biases, i.e., simple rules such as “copy the majority” or “copy prestigious individuals,” a central topic in cultural evolutionary research, might influence cultural transmission in the digital age, and conversely how digitally-supported cultural transmission might disrupt these biases. I will explore at some length two of these biases, related to prestige and popularity. Second, I will examine how cultural evolutionary dynamics could be influenced by the fact that digitally-supported cultural transmission allows virtually error-free propagation of cultural traits. I will conclude suggesting that the cultural evolution framework places the digital age in a broader context, and I will discuss how this theoretical and historical “long view” could help us to better understand the changes we are confronted with in our society.

## 2. Cultural evolution

Cultural evolution is a relatively recent scientific field that studies human and, partly, non-human cultural behavior (see Mesoudi, 2015, for a recent review). Cultural behavior is generally defined as behavior transmitted through social learning, as opposed to individual learning or genetic inheritance (Henrich and McElreath, 2003). The distinction between cultural and non-cultural behavior is not a sharp one (Morin, 2015) but it works quite well for practical purposes. Cultural evolutionists study things such as the evolution of uniquely human forms of cooperation (Boyd and Richerson, 2009; Turchin et al., 2013), indigenous knowledge of plants' properties (Reyes-Garcia et al., 2008), the cultural evolution of language (Tamariz et al., 2014; Kirby et al., 2015), the spread of fashions in contemporary culture, using cases like baby names (Bentley et al., 2004) or dog breeds (Ghirlanda et al., 2013, 2014), or how ineffective medical treatments can nonetheless be successful (Tanaka et al., 2009; de Barra et al., 2014; Miton et al., 2015), just to give a few examples. Similarly, a wide range of methodologies are used, including simulation and mathematical models (Acerbi et al., 2009; Kempe et al., 2014; Smaldino and McElreath, 2016), laboratory experiments (Caldwell and Smith, 2012; Derex and Boyd, 2015; Muthukrishna et al., 2015; Schillinger et al., 2016), phylogenetic analysis (Fortunato and Jordan, 2010; Tehrani, 2013; Watts et al., 2015), ethnographic research (Mathew and Boyd, 2014; Colleran and Mace, 2015), and comparative studies of social learning in humans and other animals (Whiten et al., 2009; Dean et al., 2012; Reindl et al., 2016).

What brings together all these researches is, more than a unitary view about how culture should be considered an evolutionary process (see Claidière et al., 2014; Acerbi and Mesoudi, 2015; Lewens, 2015, for a general discussion), a strong commitment to provide explanations that are naturalistic and quantitative, as well as grounded in cognitive science and evolutionary theory. At the minimum, all cultural evolutionists share the idea that a cultural phenomenon is a population-level aggregate of individual-level interactions and that, to explain the former, one needs to take seriously the latter. Accordingly, the works of Cavalli-Sforza and Feldman (1981) and Boyd and Richerson (1985) are considered as establishing modern cultural evolution. These works consisted in mathematical models, inspired by population genetics, developing formalisms to link micro-processes of transmission—like different “directions” of transmission, e.g., from parents to offsprings, between peers, etc. or different transmission biases, see below—to macro-processes of cultural change—like the diffusion dynamics of cultural traits. In parallel, cognitive anthropologists such as Sperber (1985, 1996) started to consider in depth the role of individual cognition in the explanation of cultural patterns, focusing on the fact that the success of some widespread beliefs may depend on them being generally attractive to human minds (I will discuss some examples in the next sections).

The psychology of digital media, in particular online activities (sometimes described as “cyberpsychology” Attrill, 2015) is a growing field (see e.g., Wallace, 2001; Suler, 2015). A cultural evolution approach adds, as mentioned, an explicit interest for the micro-macro link, in other words, for how individual-level properties (e.g., psychological) influence population-level dynamics and vice versa. In addition, the naturalistic and quantitative framework provided by cultural evolution seems perfectly suited for the study of contemporary digital media. One of the opportunities that the widespread diffusion of digital media offers to social sciences is the availability of vast amounts of data on human behavior (Lazer et al., 2009). While the understanding offered by ethnographic (e.g., Boyd, 2014) or critical-theory-inspired (e.g., Fuchs, 2014) perspectives remain clearly important, the cultural evolution approach is in a better position to make sense also of the quantitative data that digital media usage quasi-automatically produces. On the other side, computer scientists and physicists had promptly made use of these data to study the diffusion of information in digital social networks (see Weng et al., 2012; Adamic et al., 2014; Cooney et al., 2016; Del Vicario et al., 2016, for few recent examples). These works importantly include quantitative analysis and models, and they can offer valuable insights on online activity. However, the perspective of cultural evolution can complement this thread of research by providing a refined view of the micro-processes of transmission and of the psychological motivations underpinning them.

To sum up, cultural evolution may offer a privileged perspective to look at digital media, including both a sophisticated view of human behavior and a methodological attitude to modeling and quantitative analysis. In the next sections I will try to substantiate this claim with some examples of investigations that a cultural evolution approach suggests.

## 3. Transmission biases in the digital age

For the majority of cultural evolutionists the widespread utilization of social learning is the reason of the ecological success of the human species (Henrich, 2016). Social learning provides a shortcut to long and potentially dangerous individual learning and a fast and flexible alternative to genetic evolution. However, simply copying from others can be risky: to be effective, social learning needs to be selective (Laland, 2004). According to this view, social learning is made possible by domain-general heuristics—often referred to as “transmission biases” or “social-learning strategies”—helping us to choose what, when, and from whom to learn (Boyd and Richerson, 1985). To use a mundane example, imagine you find yourself in a new and unknown town, searching a restaurant for dinner. You may first decide that is worth to look to what others do, instead of trying to figure it out by yourself (“copy when asocial learning is costly”), and then that it does not make much sense to follow the first person you see in the street, but look for restaurants that seem full of customers (“copy the majority”). After few days, you might have found your favorite place, and you can stop to check where other people go (“copy when uncertain”).

Transmission biases are a good place to start as much research has been developed in cultural evolution on this topic. Theoretical models and simulations have explored the adaptive value of different biases, and predictions from the models have been tested in empirical settings (see Rendell et al., 2011, for a review). In parallel, various works have attempted to detect the presence of transmission biases in real-life cultural dynamics (e.g., Reyes-Garcia et al., 2008; Henrich and Broesch, 2011; Kandler and Shennan, 2013; Acerbi and Bentley, 2014). Importantly, for our focus on digital media, transmission biases are considered a suite of psychological adaptations shaped by natural selection (Henrich, 2016), hence generally effective in the social and physical environment of small-scale societies. A question only partially explored in cultural evolution is how these biases scale in contemporary, complex, societies, and especially in the novel digital environment.

### 3.1. Prestige

Various heuristics are available when choosing from whom to copy. From an evolutionary point of view, for example, kin share a common genetic interest, so they will be willing to circulate useful information. Copying from parents and from other close members of the family makes thus perfect sense. Elders, especially in small-scale and slow-changing societies, have two important qualities. First, they had time to learn themselves a substantial part of the cultural repertoire of the society, and, second, they must have done it effectively, exactly because they arrived to old age. Age-biased social learning is thus another evolutionary expected strategy (Henrich, 2016).

However, for specialized expertises (i.e., only few people possess them), or for expertises that exhibit variability in a population (i.e., some people are very good at them and others are not), kin- and age- based strategies are not particularly effective. In these cases, an alternative is to try to assess directly the ability of others. Copying skilled or successful individuals is then another of the heuristics suggested by cultural evolutionists (see e.g., Mesoudi, 2011, for an experimental approach). This strategy presents, in turn, another problem. Skills can be opaque, difficult to recognize, and this is especially true when one does not possess the expertise in question, which is exactly the case when there is the need to learn it. Similarly, success can be volatile, or due to luck. How many successful hunts an apprentice hunter should assess before deciding to copy from a particular individual and not from another?

A possible solution is prestige-biased social learning. Cultural evolutionist Joe Henrich defines prestige cues as a “second-order cultural learning” (Henrich, 2016, p. 45): one can make use of signs of deference, respect, or simply check from whom other people are learning, and choose those individuals as cultural models. The risk, with prestige-biased social learning, is that prestige and skills may not correlate. What if an individual is prestigious because of his hunting abilities, but I am attempting to learn how to build harpoons? What if an individual is prestigious because he belongs to an influential family, but he does not possess any particular skill? The answer is that in small-scale societies this is a minor problem. Specialization and inequality are limited, so that respected individuals will indeed be, on average, generally skilled.

Of course, the situation is different today. Our reliance on celebrities, for example in advertisement, is generally considered a good candidate for a cultural evolutionary mismatch (Henrich, 2016). The acting abilities of George Clooney are unlikely to correlate with his expertise in coffee-tasting, still, the story goes, the success of a Nestlé brand of coffee depends on the presence of the actor in the advertisements. Internet and in particular social media would possibly push things even further, because the rapidity of communications and of the extension and the number of the virtual communities. The real risk for the society is not much that we end up to parrot the—alleged—favorite coffee brand of celebrities, but that social media users will attempt to copy skills that are not existent at all (such as Clooney's coffee tasting ability) or existent, but not relevant in the local environment (such as Clooney's acting ability). More worryingly, extremist groups could make use, consciously or not, of prestige-biased influence mechanisms for on-line proselytism (Barkow et al., 2012)^2^. These ideas could be tested empirically, but, to my knowledge, not much research has been done yet. One could examine whether usage of internet and social media correlates with higher preferential attention to “global” cues of prestige (as opposed to “local” ones), possibly taking into account confounding factors such as the exposition to traditional mass-media, like television or cinema. In addition, attention to global cues of prestige does not need to be harmful, especially in a fast-changing and deeply interconnected society. Although, it might be argued that acting abilities are not necessarily relevant, the same digital media allow to access also to prestigious surgeons, programmers, or philanthropists in a way that would not be possible in a local environment.

Research on social media “influencers” is in its infancy, and results are not conclusive (see Bakshy et al., 2011; Aral and Walker, 2012, and the studies reviewed therein). Bakshy et al. (2011), for example, measured how links to webpages posted in Twitter spread in the social media itself, and found that, indeed, users with more followers and who have been already influential in the past tended to produce larger “cascades.” However, it is not clear how to distinguish the fact that the number of followers is a sign of prestige, in the cultural evolution meaning, from the fact that, at the same time, it indicates how many individuals are exposed to the link. In this sense, the effect could be simply due to a larger number of possible events of transmission. Even not considering this confounding, Bakshy et al. (2011) comment that, given that cascades-sizes are power-law distributed (i.e., there are very few large cascades, while the majority of links are never reposted), “individual-level predictions of influence nevertheless remain relatively unreliable.” They thus proceed to analyse the contribute of the actual content of the links tweeted, showing that content independently rated as more interesting and positive generated larger cascades. These findings resonate with theoretical results showing that wide-ranging events of diffusion of traits in networks are favored less by influencers than by the presence of large masses of easily influenceable individuals (Watts and Dodds, 2007).

The same celebrity influence is, at least in cultural evolution literature, mainly anecdotal, and marketing studies show that the effect of celebrities in advertisements is mediated by various cues, such as their relationship with the product advertised (see e.g., Kelting and Rice, 2013). We do not know, for every George Clooney, how many advertisements with celebrities did not succeed (Stephen Hawking, for example, was featured in the early 2000s in a high-profile campaign for an online fund platform that closed in 2004), and how many campaigns succeed without the presence of a celebrity. Moreover, as the results from Bakshy et al. (2011) suggest, there is an interaction between content and prestige. An interesting possibility is that relatively low-cost alternatives, like which coffee brand to choose or which haircut, could be celebrity-biased, but the effect would be less important for high-cost choices. This would mean that prestige-biased epidemics of extremism might not be such a realistic danger. On the other side, Clooney would not be probably able to persuade smokers to quit, for example.

In sum, although we have some convincing evidences of the effect of prestige-biased social learning in small-scale societies (Henrich and Broesch, 2011) and from laboratory experiments (Atkisson et al., 2012; Chudek et al., 2012), the question of how automatic is the influence of digital media's “influencers” in contemporary society remains open. Morin (2015) writes of “flexible imitators” that selectively use social—such as prestige—or asocial cues, depending on various factors, e.g., the above mentioned cost of the alternatives. Others (Heyes, 2016b) suggest that, at least in some circumstances, human social learning strategies are explicitly metacognitive. This means that these strategies include adjustable learning targets, changing from situation to situation, such as “copy digital natives,” referring to copying knowledgeable young persons in the specific domain of technology, instead of a general rule “copy young individuals” (Heyes, 2016a).

In this case, like in the others we will explore in the next sections, the cultural evolutionary approach suggests a perspective from which to look to digital media and a series of questions that might be addressed in further research. What is the difference between the usage of prestige cues in small-scale societies and in our contemporary digital environment? What are the differences between local prestige, as in the case of small-scale societies or in contemporary circles of friends, and global prestige, as in the case of celebrities? Is prestige modulated by content? We already mentioned a possible difference between high-cost and low-cost choices; another could be related to the presence or absence of previous knowledge: real coffee connoisseurs might be less impressed by Clooney's approval.

### 3.2. Popularity

A similar way of reasoning can be applied to frequency-dependent biases. In the idiom of cultural evolution, frequency-dependent biases are heuristics that make use of the estimated frequency of a cultural trait to help deciding whether to copy it or not. The usefulness of positive, i.e., preferences for popular traits, frequency-dependent biases is easy to understand. When in a new environment, or when confronted with a new technology, it makes sense to take advantage of the cumulative experience of other individuals.

When cultural evolutionists talk about positive frequency-dependent biases, they generally refers to “conformity” in a precise and quite restrictive sense, meaning a disproportionate tendency to copy from the majority (Boyd and Richerson, 1985). This means that, returning to our restaurants example, if 60 people are eating in restaurant A and 40 people in restaurant B, the probability to choose A should be *higher than 60%* in conformist-biased social learning. In fact, it has been noted that, in almost all cases, social learning imply to “follow the majority” in a loose sense (Boyd and Richerson, 1985). In the above case, for example, one individual would still be more likely to go to restaurant A without any particular bias, i.e., copying randomly (imagine to ask to a random person where she was for dinner and follow her advice: your probability to go to restaurant A will be 60%).

This over-response to frequency information (Efferson et al., 2008) has a special importance for cultural evolution. First, it has been shown to contribute to maintain culturally homogenous groups, despite certain levels of migrations and individual variations (see e.g., Boyd and Richerson, 2009). Second, it allows to directly “jump” to the best alternative in presence of noisy information (Henrich, 2016). In what follows I will thus use the more generic term “popularity bias” to indicate that the perception of something as popular makes it preferable to other—less popular—cultural traits, and I will reserve the usage of the term “conformity” for the technical sense described above. Finally, “social influence” simply means that people copy, without any bias, the choices of others.

As in the case of prestige, it is important to draw an explicit comparison between the conditions in which a psychological bias implementing a preference for popular cultural traits could have evolved and today's digital age. The first interesting aspect is that, in a small-scale and perhaps illiterate society, popularity needs to be estimated from various cues. The situation with digital media appears clearly different. Popularity is quantified and explicitly made public—the number of Facebook “likes” or “share,” the number of Twitter “retweets,” etc.—in practically all digital platforms. While one could speculate whether the success of this practice might be due to a universal sensitivity to this kind of information, as a cultural evolution perspective would suggest, it is not clear what kind of effect this could have on cultural transmission patterns. One possibility is that such low-cost availability of popularity signals would discourage individual exploration, prompting people to follow cheap social cues (Derex and Boyd, 2015), with digital media amplifying the effect of popularity-biased cultural transmission.

For example, success in digital media, especially regarding internet websites, has been repeatedly described as following a power-law distribution (as mentioned in the previous section for the links posted on Twitter). Power-law distributions are typical of winner-take-all markets, with very few websites monopolizing visitors whereas the vast majority remains relatively unsuccessful (Adamic and Huberman, 2000). However, it is useful to remind that power-law distributions are not necessarily generated by popularity-biased dynamics, as defined above. Power-law distributions naturally arise with unbiased social influence, because simply copying at random amplifies small initial differences. In fact, cultural evolutionary studies have shown that power-law distributions are present in many domains where social influence is important, such as baby names, dog breeds, scientific citations (Bentley et al., 2004), or even decoration styles in neolithic pottery (Neimann, 1995), where one can safely exclude the influence of digital media. The tell-tale of a positive-frequency-dependent bias is a distribution that is even more skewed in favor of successful items than power-laws (Mesoudi and Lycett, 2009).

In addition it is difficult, when not impossible without additional data, to set apart the effect of social influence and the effect of the intrinsic quality of the items in creating these skewed distributions (Aral and Walker, 2012; Muchnik et al., 2013; Morin, 2015). Ghirlanda et al. (2013), trying to deal with this problem, examined the case of dog breeds popularity. They showed that desired characteristics of breeds, such as trainability or good health, were not correlated with their success. This suggests that, in this specific domain, the role of popularity, or simply social influence, is more important than the intrinsic characteristics of the cultural traits, i.e., the dog breeds themselves.

Some studies manipulated directly the perceived popularity of items in digital media, trying to detect the effect on their subsequent success. In a recent experiment, Muchnik et al. (2013) assigned randomly more than 100,000 comments submitted to a website with a structure similar to Reddit to three treatment groups: up-treated (comments were artificially given a +1 rating at their creation), down-treated (comments were artificially given a −1 rating at their creation), and control. Up-treated comments were indeed more likely to be subsequently up-voted than control. Down-treated comments were, as expected, more likely to be subsequently down-voted than comments in the control group. However, they were up-voted to a greater extent, so that the net effect was slightly positive, even if not significant with respect to the control group, as if users of the website tended to counterbalance negative comments. Muchnik et al. (2013) explain their results as due to an increasing turnout (i.e., up- or down-treated comments generated overall more ratings than comments in the control group) coupled with a common preference for positive ratings.

In a previous large-scale experiment, Salganik et al. (2006) created a digital “artificial market” where subjects could listen to and download unknown songs. Participants in the social influence condition could see how many times a song was downloaded previously, and they were randomly assigned to one of eight “worlds” where the counts of download were evolving independently. Salganik et al. (2006) showed that the social influence condition created more inequality (defined as difference between successful and unsuccessful songs) and unpredictability (defined as the difference between songs' results in the different worlds) with respect to the independent condition, where participants did not have information on previous download. Interestingly, two forms of visual presentation were proposed to participants in the social influence condition: in the first, the songs were presented in the same configuration of the independent condition, simply adding the number of previous downloads, and in the second they were presented as an ordered list, with the most downloaded on the top. Social influence was noticeably stronger in the latter case (more on this below).

Unpredictability, however, was not complete: there was a significant correlation between the perceived quality of the songs, as measured in the independent condition, and their success in the social influence condition or as Salganik et al. (2006) put it: “in general, the “best” songs never do very badly, and the “worst” songs never do extremely well.” Given that choices (downloading or not a song) were extremely low-cost for the participants and the fact that the songs were previously unknown, the effect of popularity seems relatively limited in this experiment (Lewens, 2015; Morin, 2015, argument more thoroughly for a similar interpretation of these results). In a follow up study, the manipulations were stronger, such as completely reversing the perceived popularity order of the songs, i.e., presenting as the most popular the “worst” song of the independent condition, and so on (Salganik and Watts, 2008). Again, however, the best songs tended to recover their popularity in the long run. Moreover, strong distortions of the correlation between intrinsic appeal and popularity were intuitively perceived by the participants, as showed by the fact that they resulted in fewer downloads overall. As above, the effect of popularity seems to be more nuanced that what an intuitive, clear-cut, understanding would suggest.

A more extreme version of the explicit advertisement of popularity cues is the proliferation of “top-N” lists. The spreading of top-lists predates digital media, and it is almost an hallmark of the broadcast era (in the United Kingdom the first introduction of a top-chat program in BBC radio dates back to 1957^3^), but it reached enormous diffusion in the recent years, with on-line top-lists of virtually everything. From a cultural evolution perspective, top-lists are not only sources of cheap estimates of popularity, but they also supply a direct way to implement a variant of the above mentioned conformist-bias, giving disproportionate publicity to already popular items (Acerbi and Bentley, 2014). The presentation of alternatives in form of top-lists, or ranked tables, do seem to enhance popularity influence (Salganik et al., 2006).

Another, more elaborate, variant of popularity displays is represented by the spreading of information in form of consumer—as opposed to “expert”—reviews, whether as a part of commercial websites (such as Amazon), or through websites specifically dedicated to reviews (such as Tripadvisor, Yelp, etc.). The positive economic effect of favorable reviews has been shown in several domains, including books (Chevalier and Mayzlin, 2006), restaurants (Luca, 2011), or hotels (Ye et al., 2009). The where-to-go-to-dinner example I used to illustrate cultural transmission biases looks rather outdated nowadays, when people can glance at their smartphones and obtain cheap, real-time, information on all restaurants in their surroundings. Finally, a large number of websites and, in particular, almost all social media and commercial websites, provide direct personalized recommendations, e.g., “inspired by your browser history" in Amazon, “who to follow” in Twitter, etc.

Consumer reviews and recommendation systems have complex effects on users' preferences (Duan et al., 2008; Fleder and Hosanagar, 2009) that is not possible to explore in this article. Moreover, the contemporary trend might even be to replace these explicit systems with more subtle presentation cues, embedded in the layout of the user interface, or simply deciding the informations that are presented and the informations that are not, as in Facebook News Feed (Vanderbilt, 2016). These recent and less recent (such as top-lists diffusion) developments are stimulating material for future cultural evolutionary studies, and looking at them through the perspective of cultural transmission biases seems a promising direction.

In conclusion, the details of how popularity influences the spreading of cultural traits need further investigation. The quantitative data resulting from digital media usage may be of great significance for this endeavor. At the same time, new ways to signal and perceive popularity in the digital environment represent an important new area of research for cultural evolutionary studies.

## 4. Preservative and reconstructive cultural transmission

How faithful is cultural transmission? While, in the popular image, cultural “evolution” implies that ideas and behaviors spread by replicating gene-like from individual to individual, practitioners tend to be more cautious about the analogy genes-cultural traits, in particular regarding fidelity of transmission. The term “meme,” invented by Richard Dawkins, is dismissed by the majority of cultural evolutionists, even though sometimes used in social-media literature (e.g., Weng et al., 2012; Adamic et al., 2014).

The oral transmission of stories provides a case in point. Transmission chain experiments, where individuals are asked to iteratively listen to and repeat short narratives (starting from Bartlett, 1932), have shown that, because of memory and attention limits, or biases from previous knowledge, the original material is quickly disrupted (more on transmission chain experiments below). In fact, what is surprising is on the contrary how some orally transmitted folktales have remained relatively stable through centuries or even millennia (Graça da Silva and Tehrani, 2016).

There are various options to explain cultural macro-stability. Some (see e.g., Sperber, 1996; Sperber and Hirschfeld, 2004; Morin, 2015) prefer to concentrate on universal, or slow-changing, factors of attraction that make some cultural traits, or some features of them, particularly memorable, or more likely to be reproduced individually. The stability of a long, oral, transmission chain of a story—say Cinderella—does not depend on a series of faithful acts of copying, but on the fact that some features of the story are particularly likely to be remembered and reconstructed in successive retellings (the example of Cinderella is used in Acerbi and Mesoudi, 2015). The Pumpkin Coach might be one cultural attractor, as an example of a minimally counterintuitive concept (a concept that mainly fits our intuitive cognitive expectations but with few exceptions; for an analysis of the success of folktales due to the presence of minimally counterintuitive concepts see Norenzayan et al., 2006); another might be the relationship between Cinderella and the wicked stepmother (stepparents are considered a serious threat for stepchildren from the point of view of kin selection theory, see Daly and Wilson, 1999).

Others links instead macro-stability to precision of transmission at individual level (micro-stability). Some focus on the fact that, compared to other species that make nevertheless use of social learning, such as other great apes, humans are faithful copiers (Tennie et al., 2009; Dean et al., 2012). Another possibility is that the above mentioned transmission biases provide a way to repeatedly encounter the same behavior, supplying redundancy to the process of cultural transmission (Boyd and Richerson, 1985). Finally, another option yet is provided by epistemic technologies (Sterelny, 2006), i.e., modifications of the external environment that improve individuals' cognitive abilities, in this case specifically related to facilitate transmission, including extensive apprenticeship or practice.

Acerbi and Mesoudi (2015) argued that these explanations are not mutually exclusive, and that their importance varies depending, among other things, on the domain being studied. Some cultural domains, such as orally transmitted stories, can be considered mainly based on reconstructive cultural transmission, i.e., they derive their stability from the presence of features that are likely to be reconstructed each time by individuals, no matter how faithful is the process of transmission itself. Other domains, for example complex technologies, are characterized by preservative cultural transmission, implemented through faithful copying and external epistemic technologies. As might be expected, reconstruction and preservation, or attraction and faithful copying, are important, in various degrees, in all cultural domains. Rhymes are epistemic tools that make attractive stories even more transmissible (Rubin, 1995); recipes books contain scripts that make universally palatable dishes easier to prepare (Acerbi and Mesoudi, 2015).

Digital media can therefore be considered as a technology that makes cultural transmission more preservative. Cinderella does not need to be listened to, remembered, and retold, but can be “shared” in social media, and practically replicated with extremely low mutation rate. In this sense, the usage of the term “meme” for content that spreads in digital media could be possibly reconciled with its meaning in cultural evolution. An interesting question, from a cultural evolution perspective, is whether the degree of fidelity of transmission influences the kind of content that is more likely to spread.

Cultural evolutionists have investigated content effects experimentally mainly using the above mentioned transmission chain methodology. Transmission chain experiments show that the distortion of the content are consistent, that is, some kinds of content tend to survive along the chains, and others do not. A growing, if somehow unsystematic, catalog of so-called content biases is being built, including among others: a bias for social information (or gossip), involving peoples' relationships and interactions (e.g., Mesoudi et al., 2006); a bias for survival-relevant information, such as location of resources or predators (e.g., Stubbersfield et al., 2015); a bias for content that elicits emotional reactions, especially related to disgust (e.g., Eriksson and Coultas, 2014); a bias for the above mentioned minimally counterintuitive concepts (e.g., Barrett and Nyhof, 2001); a negativity bias, where negatively valenced information is preferred to positively valenced one (Bebbington et al., 2017); a bias for simplicity in linguistic structure (balanced by informativeness, e.g., Kirby et al., 2015), and so on.

However, what if information can be easily reproduced with high-fidelity, as it happens in preservative digital transmission? Promising steps in this direction have recently been made, for example, by experiments from Eriksson and Coultas (2014) and Stubbersfield et al. (2015), which considered each passage in the transmission chain as composed by three distinct phases: choose-to-receive, encode-and-retrieve, and choose-to-transmit. The choose-to-receive and the choose-to-transmit phases indicate respectively the willingness to receive and to circulate cultural information. They are comparable to social media “share,” as they do not require the memorization and the repetition of the material, which are required only in the encode-and-retrieve phase. Eriksson and Coultas (2014) found that the bias favoring disgust-related information was operating in the same way in all phases of the transmission. Stubbersfield et al. (2015) compared social and survival information biases, and they found that social information bias had an advantage on survival information bias only in the encode-and-retrieve phase (i.e., the “standard” transmission chain methodology), but not in the choose-to-receive and choose-to-transmit.

Berger and Milkman (2012), with a different approach, examined directly what people share in a 3-month “field study” conducted on *New York Times* articles. Among other findings, they report that the most shared articles were characterized by a preponderance of positive emotion-valenced terms with respect to negative emotion-valenced ones. This might appear surprising when compared with transmission chain studies that found, on the contrary, that a story with negative content had an advantage in terms of probability to spread and to not be distorted (Bebbington et al., 2017). This negative bias, in terms of favoring attention and memorization, has been confirmed in several experiments, and there are evolutionary reasons to think that negative information should be more salient than positive one (Fessler et al., 2014). One way to reconcile these findings with the results of Berger and Milkman (2012) might be indeed to consider that they studied a paradigmatic case of digitally-mediated preservative transmission, whereas the findings supporting the importance of a negative bias come from cases of reconstructive transmission, or simply related to recall. In this particular case, digital media would favor—because memory and reconstruction are less important than, perhaps, self-presentation motifs, and desire to share positive content with familiars and friends—different content with respect to traditional oral transmission. Other features, for example simplicity and repetitiveness, which have been shown important for the maintenance of oral traditions (Rubin, 1995), seem to contribute in the same way to the success of digital content (Shifman, 2012).

Interestingly, some social media texts, in particular Facebook updates, come with the explicit instruction to “copy-and-paste”—as opposed to share—them. It is not entirely clear why this is the case^4^, but, from the point of view we are discussing, copy-and-paste reintroduce variation in highly preservative digital transmission, allowing for modifications that could make the messages more successful (Acerbi and Mesoudi, 2015). Adamic et al. (2014) estimated a “mutation rate” of μ = 0.11 for Facebook status updates asked to be copy-and-pasted, i.e., 11, every 100 copies, were different from the original, which is extremely high considering the fidelity provided by the digital support.

In fact, some researchers (for example Shifman, 2013) have proposed that one of the main features of internet “memes” is to provide templates that individuals use to introduce personal innovations. Whereas, in oral transmission reconstruction is practically unavoidable, in digitally-supported transmission the content is actively modified by individuals. Shifman (2013) distinguishes two major ways individuals use to modify content: “remix,” involving the digital editing of pre-existent material, and “mimicry,” involving the actual creation of a new content, inspired by the source. A well-known example of remix is the “Hitler Reacts” meme^5^, where fake subtitles, often related to contemporary popular culture topics, are added to a scene of the 2004 movie “Downfall,” where an angry Hitler addresses his strict collaborators in his bunker few days before committing suicide. An example of mimicry is instead “Harlem Shake.” In the first 2 weeks of February 2013 around 40,000 videos, in which groups of people dance on the music of the song “Harlem Shake,” were uploaded to YouTube^6^. The videos are all based on the same concept: they usually start with a single person dancing, surrounded by other people apparently indifferent to the event. Suddenly, the entire group starts to dance, generally with exaggerated and spasmodic-looking movements, often using props and costumes.

More studies are needed to clarify whether there is a specific effect of digital media on the content that is transmitted, but, again, cultural evolution may provide a favorable perspective to investigate this problem. In addition, the distinction preservative/reconstructive is only one of the possible ways to look at the effects of supporting cultural transmission digitally. It has been argued, for example, that universal factors of attraction, or stable content-biases, are especially important with respect to context-based transmission biases (such as popularity and prestige, examined in the previous sections) when cultural transmission chains have two properties. First, they extend through long time-scales, and, second, they are “narrow,” that is, the connections between individuals are sparse (Morin, 2015). Digital media seem exactly to be the opposite case, providing fast spreading and high connectivity between individuals (Doer et al., 2012). On the other side, successful cultural traits that spread through digital media can reach enormous diffusion—the well known Gangnam style music video has, as of September 2016, more than two and half billions views on YouTube^7^—which may imply they can reach a very diverse audience, possibly by tapping common psychological preferences.

As above, this review of the cultural evolution literature suggests a way to frame possible questions, more than providing answers. Does the fact that digital media support cheap and high fidelity transmission have an influence on the kind of content that is more likely to spread? What is the role of mechanisms that introduce variation in digital transmission? Are universal cognitive biases more, less, or equally important in the digital age?

## 5. Taking the long view

Overall, very few studies in cultural evolution have dealt with these subjects. As a consequence, this review is only proposing some possible directions, and, mainly, suggesting that cultural evolution can provide a “long view” to the contemporary digital environment. When put into perspective, the new phenomena that characterize our digital age appear to have their roots in deeper psychological and historical dynamics, and, to understand what is genuinely new and what is not, we may need to take seriously these dynamics.

The spread of *massive digital misinformation*, for example, is considered one of the most worrying contemporary global risks by the World Economic Forum^8^. Models that explicitly address the spread of misinformation in social networks (Acemoglu et al., 2009; Del Vicario et al., 2016) could greatly benefit of the inclusion of the knowledge developed in cultural evolution. The transmission chain experiments mentioned in the previous section show that certain kinds of information, related for example to gossip or disgust, are more likely to spread than others. How these, and others, predispositions to be influenced in cultural transmission interact with the novel characteristics of digital media (such as high fidelity of transmission, speed, etc.) is material for future studies.

A similar reasoning can be applied to another allegedly worrying phenomenon associated to digital, in particular social, media, that is, the formation of echo chambers. The term “echo chambers” describes the fact that individuals tend, in social media, to associate in communities of like-minded people, and they are thus repeatedly exposed to the same kind of information (e.g., a political ideology) and, especially, they are not exposed to information that could counterbalance it. More concerning, it has been suggested that groups of like-minded people tend to produce opinions that are not an “average” of the opinions of the members of the groups, but their radical version, according to a phenomenon called “group polarization” (Sunstein, 2002).

The empirical evidence for the existence of echo chambers in social media is, however, mixed. Studies showing their existence considered explicitly separated communities of individuals (e.g., Facebook users associated to groups coded as “science news” and “conspiracy theories” in Del Vicario et al., 2016), whereas other researches gave a more nuanced image. Barberá (2014), in a study of Twitter accounts from Germany, Spain, and the United States, found that the usage of social media decreases political polarization, arguing that social media contains more weak ties (i.e., acquaintances or occasional contacts as opposed to close friends or family) with respect to offline networks. In another example, Shore et al. (2016) found that Twitter users post links that are, on average, more moderate than the links they receive in their feed, and that the perception of polarization at global level is due to the activity of a core of few, but more active, extremist users.

As above, a cultural evolution approach suggests to look at polarization, and echo chambers formation, from a broader perspective. Cultural evolutionists have identified, among the cultural transmission biases discussed in the previous sections, one that refers to “self-similarity,” i.e., to the fact that individuals preferentially copy from others similar to them. This has been particularly studied for the arbitrary signals that mark ethnic groups membership. As in the case of prestige bias, or popularity bias, there are reasons to think that a self-similarity bias is an adaptive strategy. The logic is that people of the same group are more likely to live in similar situations, and thus to share the same challenges (Henrich, 2016). One may thus wonder whether or not social media are amplifying the effects of the similarity bias with respect to offline interactions. How polarized are groups of offline friends or coworkers? And what about traditional, broadcast, media?

The broad perspective suggested by cultural evolution does not imply, of course, that the recent modifications produced by digital media are not important, or that media are neutral, and they do not influence what is transmitted. On the contrary, the long view proposed here might be necessary to bring out clearly the novelties. An example toward this direction concerns the incredible amount of user-generated content that has been developed and published with the advent of the so-called Web 2.0, such as blogs, videos, or wiki platforms (van Dijck, 2009). If the motivations of producing some of this content, for example in the case of blogs or video sharing, are likely to be self-promotional, other collaborative enterprises (e.g., Wikipedia, or the WikiHow platform) are more puzzling from a cultural evolutionary point of view. It is common, in cultural evolution (starting from Rogers, 1988), to consider social learners as “information scroungers,” that do not pay the cost—and avoid the risk—of individual trial-and-error, relying on the effort of individual learners (Rogers' model shows that populations composed by only, or a great majority of, social learners can not track environmental variation). However, digital media made obvious that, if they have the possibility, individuals seem to be happy to provide, for free, information to unknown “scroungers.” How, and to what degree, this may provide a return in terms of reputation or within-group advantage is an interesting question for cultural evolutionary studies of digital media.

Finally, digital media interactions involve substantial changes in the form in which information is transmitted. On one side, digital media favored a surge of text-based, as opposed to oral, communication. For example, the majority of day-to-day conversations between US teenagers happen through text messaging. Non-digital, in person, contacts are in fourth position, preceded by instant messaging and interactions through social media websites^9^. Arguably, previous works on the differences between oral cultures and cultures where writing is widely diffused (e.g., Ong, 1982) are an intriguing starting point to shed light on this phenomenon. Ong (1982), for instance, classified (his) contemporary culture as characterized by a “secondary” orality, i.e., the orality promoted by traditional-broadcast media, profoundly influenced by writing and thus different from the primary orality. One could use the term “secondary literacy” to describe the current situation. Secondary literacy provide, as primary literacy, a way to improve micro-stability of transmission, making it highly preservative, as discussed at length in the previous section. However, it also differs from primary literacy in several respects, including, among others, a more widespread utilization, informal tone, and instantaneity of transmission. In parallel, transmission based on digital media is characterized by the facility of including non-written content, such as images and videos. A significant proportion of the content successfully spreading in the digital environment is in fact characterized by a combination of visual and textual features (think, for example, to image-macro“memes” such as *LOLcats*, or “demotivational” posters^10^).

## 6. Conclusion

In the previous sections I highlighted few of the possible investigations that a cultural evolution approach to digital media suggests. One is to look to how traits spread in digital media through the lens of cultural transmission biases. Transmission biases, such as preferentially paying attention to prestigious individuals, or to items that are already popular, are considered adaptations. As such, they are tuned to the conditions of small-scale, slow-changing, and orally-based, societies. How these transmission biases operate in contemporary culture, in which cultural transmission heavily relies on the support of digital media, is an important, and so far unanswered, question. In the same time, I endorsed an elastic view of these biases. Popularity and prestige are not—or, at least, not always—blind forces that push people to copy compulsively. Fears of internet epidemics of extremism, harassment, or similar, driven by influentials or informational cascades, should be considered in a broader context. The quantitative data produced by digital media, together with dedicated experiments, may help us to understand when and how social cues, such as prestige and popularity, interact with the individual evaluation of the content of cultural traits and with other tendencies.

Next, I examined how digital media can be seen as a technology that makes cultural transmission preservative, by providing, practically for free, high fidelity of transmission. This is quite a departure from the conditions usually examined in cultural evolutionary experiments, where items are generally transformed when passing from an individual to another. In addition, digitally-mediated cultural transmission is characterized by other features such as speed, dense connections among individuals, heavy utilization of writing and, in the same time, facility of combining written and audio-visual content. How the interactions of these features influence what kind of content is more likely to spread is another important investigation.

Cultural evolution is a mature field that could give its contribution to the exam of contemporary cultural phenomena. The digitalization of many instances of cultural transmission seems both relevant for our society and suitable for the theoretical and methodological tools that cultural evolutionists have developed. More empirical and modeling works are needed for this task, and possibly the suggestions sketched here may provide some guidance.

## Author contributions

AA wrote the article, conceived the work, searched and studied the literature, and elaborated the viewpoint that the article expresses.

## Funding

AA was supported by The Netherlands Organization for Scientific Research (NWO VIDI-grant 016.144312).

### Conflict of interest statement

The author declares that the research was conducted in the absence of any commercial or financial relationships that could be construed as a potential conflict of interest.

## Footnotes

^1^https://newsroom.fb.com/company-info/

^2^See also: http://www.cato-unbound.org/2016/02/08/jerome-h-barkow/how-internet-subverts-cultural-transmission

^3^https://en.wikipedia.org/wiki/Pick_of_the_Pops

^4^One reason might be that shared malicious messages or hoaxes, if reported as such by users, can be easily traced back to the original, and in case all the thread can be deleted by administrators of the social media. Each copy-and-pasted status, by contrast, is an independent piece of content, and can not be immediately linked to the others.

^5^http://knowyourmeme.com/memes/downfall-hitler-reacts

^6^http://knowyourmeme.com/memes/harlem-shake

^7^https://www.youtube.com/watch?v=9bZkp7q19f0

^8^http://reports.weforum.org/global-risks-2013/risk-case-1/digital-wildfires-in-a-hyperconnected-world/

^9^http://www.pewinternet.org/2015/08/06/teens-technology-and-friendships/

^10^http://knowyourmeme.com/memes/image-macros
